# Clinical perspectives and concerns of metformin as an anti‐aging drug

**DOI:** 10.1002/agm2.12135

**Published:** 2020-12-01

**Authors:** Chuyao Wang, Bangwei Chen, Qian Feng, Chao Nie, Tao Li

**Affiliations:** ^1^ BGI‐Shenzhen Beishan Industrial Zone Shenzhen China; ^2^ Department of Biomedical Engineering University of Rochester Rochester NY USA; ^3^ School of Biology and Biological Engineering South China University of Technology Guangzhou China; ^4^ China National GeneBank BGI‐Shenzhen Shenzhen China

**Keywords:** aging, clinical pharmacology, longevity, metformin

## Abstract

As percentages of elderly people rise in many societies, age‐related diseases have become more prevalent than ever. Research interests have been shifting to delaying age‐related diseases by delaying or reversing aging itself. We use metformin as an entry point to talk about the important molecular and genetic longevity‐regulating mechanisms that have been extensively studied with it. Then we review a number of observational studies, animal studies, and clinical trials to reflect the clinical potentials of the mechanisms in lifespan extension, cardiovascular diseases, tumors, and neurodegeneration. Finally, we highlight remaining concerns that are related to metformin and future anti‐aging research.

## Graphical abstract

In this paper, we review mechanisms and clinical studies of metformin. Papers reviewed include studies in longevity, cardiovascular diseases, cancer, and neurodegeneration. In the end, we provide insights to guide future research.

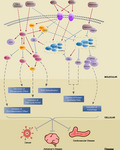

## 1 INTRODUCTION

The life expectancy in many countries is projected to exceed 85 years by 2030.^1^ Globally, one quarter of the population is expected to be in their 60s or older in 2050.^2^ Nonetheless, the unprecedented longer life expectancy heralds a staggering number of people living with age‐related diseases and considerable burdens on the social, economic, and health care systems worldwide. There is a pressing need to combat the challenges posed by age‐related diseases and increase people's health span, the amount of time that people are alive and healthy. However, while age‐related diseases can often coexist, current delivery of health services and research efforts have continued to deal with the diseases ineffectively in an insular fashion.^3^ In contrast, mechanisms that account for the phenotypes of old age, such as impaired metabolism, dysregulated immune profile, and abnormal DNA methylome, underlie many chronic diseases.^4^ Therefore, modifying the mechanisms of aging directly seems to be a more productive approach to fundamentally curb the growth of chronic diseases.

A growing number of studies have indicated that metformin, a well‐known drug against type 2 diabetes (T2DM), can potentially stall aging and delay the onset of age‐related diseases in humans. In this review, we survey a selection of articles about metformin to give an overview of the anti‐aging mechanisms that have been studied with it and the extent of their effectiveness. Then we highlight remaining questions and concerns that are preventing approved use of metformin as an anti‐aging drug and future research directions.

## 2 EXTENDING HEALTH SPAN

Almost all life forms constantly sit on a balance between production and maintenance, and under low nutrient conditions when reproduction is more challenging; in order to ensure reproductive success, increasing somatic maintenance is necessary to prolong the reproductively competent period and, consequently, lifespan.^5^ Hence, calorie restriction (CR) without malnutrition is one of the most reliable approaches to extend both lifespan and health span in various vertebrate and non‐vertebrate species. However, CR is difficult to sustain and implement, since individuals must remain in a state of hunger and endure feelings of starvation, fatigue, and irritations. Besides, individuals who practice CR are more susceptible to viral infections^6^ and resistant to wound‐healing,^7^ both of which impede its widespread use. Alternatively, metformin can act as a CR mimetic by triggering the nutrient sensing pathways that sense and respond to the changing intracellular and extracellular energy and nutrient levels without actually restricting calorie intake.^8^

**FIGURE 1:**
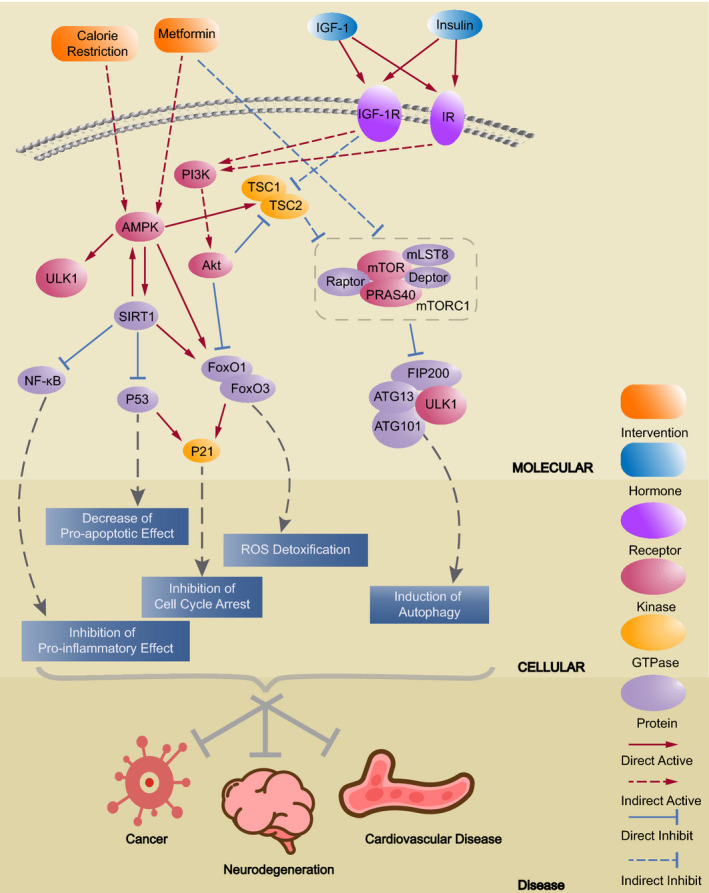
Metformin could decrease the incidence of age‐related diseases. Calorie restriction and activation of IGF‐1 receptor (IGF‐1R) and insulin receptor (IR) lead to downstream effects such as decrease of pro‐apoptotic effect, ROS (reactive oxygen species) detoxification, and inhibition of cell cycle arrest, which help protect against age‐related diseases. Metformin elicits the same effects by activating AMPK and inhibiting mTORC1

Metformin acts on the 5′‐AMP‐activated protein kinase (AMPK) (Figure 1). The activating capacity of the AMPK signaling pathway declines with aging, and its decline disturbs autophagy, increases cellular stress, and promotes inflammation, which further provokes many age‐associated diseases, such as cardiovascular disease, diabetes, and cancer.^9^, ^10^ Correspondingly, increased activation of the AMPK pathway has been shown to extend lifespan in animal models in response to CR and pharmaceutical agents, such as metformin.^11^ AMPK phosphorylates and activates Unc‐51 like autophagy activating kinase 1 (ULK1) of the ULK complex that promotes autophagy as well as activates the forkhead box O‐class (FOXO) transcription factors that transactivate the genes involved in detoxification, autophagy, tumorigenesis suppression, and energy homeostasis.^5^ Furthermore, metformin attenuates the endoplasmic reticulum (ER) stress and oxidative stress often caused by nutrition overload, aging, and reactive oxygen species (ROS) production. ER and oxidative stress contribute to chronic inflammation, and AMPK inhibits nuclear factor kappa‐light‐chain‐enhancer of activated B cells (NF‐κB), the major regulator of innate and adaptive immunity, and relieves ER and oxidative stress by promoting the expression of mitochondrial uncoupling protein (UCP‐2). UCP‐2 has an active role in inhibiting the production of reactive oxygen species (ROS) in mitochondria, suppressing the ROS produced by the reduced nicotinamide adenine dinucleotide phosphate (NAD(P)H) oxidase, and inducing the expression of thioredoxin (Trx) by activating FOXO3.^12^

Another ground for metformin and nutritional signals to interact is the gut microbiota. Microbial nutrition sensors such as the phosphotransferase system (PTS) controls the downstream transcription factor Crp in response to the nutritional landscape in the gut. Agmatine, whose level is regulated by Crp, is important for the effects of metformin by interacting with lipid and peroxisome metabolism. In *Caenorhabditis elegans*, metformin treatment decreased lipid droplet size and increased peroxisomal abundance when the Crp gene is not deleted. The group also used computer modeling to predict whether the PTS‐Crp axis is present in T2DM humans treated with metformin, and the predicted agmatine production capacity was significantly higher.^13^

Metformin is also implicated in DNA methylation, which is the process of adding a methyl group to the fifth carbon on cytosine facilitated by DNA methyltransferases (DNMT). DNA methylation usually leads to gene silencing by interacting with transcription mechanisms, and global hypomethylation and promoter hypermethylation are often observed in aged people.^14^ Metformin activates AMPK and upregulates let‐7, a family of microRNAs that bind to and degrade H19, a long noncoding RNA that is normally downregulated in adults. H19 causes aberrant methylation by binding to and inhibiting s‐adenosylhomocysteine hydrolase (SAHH), which hydrolyzes s‐adenosylhomocysteine (SAH), an inhibitor of DNMT3B.^15^

Quite a few studies have corroborated the proposed life‐extending effects of metformin. Metformin extended health span and lifespan in *C elegans* (Table 1).^16^ Furthermore, a low dose of metformin supplemented in middle‐aged male mice's diet led to a 5.83% extension of mean lifespan.^17^ A longitudinal study compared the methylation profiles of the white blood cells from 12 healthy individuals at the beginning, 10 h, and 7 days after metformin treatment and revealed 11 consistently differentially methylated sites. The differentially methylated genes included CAMKK1, a regulator of AMPK and glucose uptake, BACE2, involved in neurodegenerative disorders and insulin production, and ADAM8, which is related to monocyte mobility and inflammation‐caused diseases. As the cells were from healthy individuals, regulation of ADAM8 expression shows that metformin's anti‐inflammatory effect is independent of diabetic status.^18^

**TABLE 1:** Summary of major animal studies and clinical trials about the effects of metformin

Study	Organisms	Application Scheme	Effect
**Life extension effect**			
Slack (2012)^68^	Fruit flies	1,10,100 mM, every day	1–10 mM, no effect on survival; >10 mM, lifespan decrease Increase mean lifespan by 18%, 36%, 3%
Cabreiro (2013)^16^	*C elegans*	25, 50, 100 mM, every day	Increase mean lifespan by 14%, 6%, 0%
Martin‐Montalvo (2013)^17^	Mice	100 mg/kg, every day, started at 3, 9 or 15 months	0.1%, lifespan increase by 5.83%; 1%, lifespan decrease by 14.4%
Zhong (2017)^15^	Human	Initial dose: 750 mg/day; increased weekly up to 1500 or 2250 mg/day for 3–12 weeks	Increase in AMPK phosphorylation; Decreased tumor cell proliferation; Decreased H19 levels; Increased methylation in genes H19, DMRTA2, KCNG2, PSMD10, TRA2A
Kulkarni (2018)^82^, ^86^	Human	Metformin and placebo in either order each for 6 weeks with a 2‐week washout period in between. Patients took 1–2 metformin/placebo capsules per day	Metformin influenced genes and pathways related to metabolism, collagen, mitochondria, and DNA repair
Pryor (2019)^13^	*C elegans*	Metformin was supplemented in cell culture at 6.25, 12.5, 25, 50, 100 mM	Decreased lipid droplet size and abundance and increased peroxisome abundance
**Anticancer effect**			
Mitsuhashi (2014)^41^	Human	1500–2250 mg/day, for 4–6 weeks	Inhibited endometrial cancer cells grow in vivo
Anisimov (2011)^39^	Mice	100 mg/kg‐bodyweight starting at the age of 3, 9, and 15 months	Delayed tumor onset by 22% and 25% respectively at the age of 3 and 9 months
H. P. Chen (2013)^44^	Human	Based on the condition of patients with type 2 diabetes mellitus	Hepatocellular carcinoma risk decreases
**Reduce cardiovascular disease risk**			
Lexis (2015)^35^	Human	ST‐segment elevation myocardial infarction (STEMI) patients, 500 mg twice daily, for 4 months	Cardiovascular risk decreases
Goldberg (2017)^36^	Human	850 mg twice daily, for over 3.2 years	Coronary atherosclerosis risk decreases
Karnewar (2018)^28^	Mice	50 mg/kg‐bodyweight for 14 months	Ameliorated age‐related atherosclerosis; Significantly reduced macrophage recruitment and inflammatory cytokines
Kanamori (2019)^31^	Mice	200 mg/kg‐bodyweight per day for 4 weeks	Partially reversed left ventricular dilatation caused by δ‐sarcoglycan deficiency
Griffin (2018)^83^	Human	Up to 500 mg metformin tablets (Glucophage^®^) per day. Participants followed up to 0.99 years (SD 0.3 years)	Small improvements in glycated hemoglobin, estimated glomerular filtration rate, LDL cholesterol. Reduced vitamin B12 level
**Anti‐Alzheimer's disease effect**			
Chen (2009)^58^	Mice	2–5 mg/ml for 6 days	Both intracellular and extracellular Aβ species increases
Li (2012)^59^	Mice	200 mg kg^−1^ day^−1^ for 18 weeks	AD‐like biochemical changes decrease
Koenig (2017)^56^	Human	Metformin or placebo for 8 weeks	Executive functioning improves
**Anti‐Parkinson's disease effect**			
Katila (2017)^66^	Mice	200 mg kg^−1^ day^−1^ for 7 days	Metformin provides neuroprotection against MPTP neurotoxicity

## 3 CARDIOVASCULAR DISEASES

Cardiovascular diseases (CVDs) are the leading cause of death globally, claiming around 17.9 million lives per year.^19^ Aging is the dominant CVD risk factor.^20^ In the United States, roughly 70%–75% of people who are 60–79 years old are afflicted with CVDs.^21^

Dyslipidemia, insulin resistance, and chronic inflammation that commonly occur in older people make them more susceptible to CVDs. AMPK activation by metformin can suppress fatty‐acid desaturase (FADS) genes, reducing the circulating levels of lipid metabolites and low‐density‐lipoprotein (LDL) cholesterol.^22^ Metformin also improves insulin sensitivity, helps weight loss, and reduces perceived hunger and food intake.^23^ This can be attributed to the interaction between the GDNF family receptor α–like (GFRAL) receptor in the central nervous system and growth differentiating factor 15 (GDF‐15), whose expression is increased most prominently in the liver and gastrointestinal system by metformin.^24^, ^25^ Although metformin does not directly affect coronary artery disease through the GDF‐15 pathway, the GDF‐15 dependent weight loss effect may contribute to higher insulin sensitivity.^26^ Metformin also inhibits vascular inflammation that can lead to plaque formation by blockading the phosphatidylinositol 3′‐kinase (PI3K)‐protein kinase B (Akt) pathway and its downstream NF‐κB pathway.^27^ Furthermore, mitochondria dysfunction and endothelial senescence contribute to higher risks of CVDs, and AMPK improves mitochondrial biogenesis and function by enhancing trimethylation of histone 3 lysine 79 (H3K79) via the sirtuins (SIRT)‐disruptor of telomeric silencing 1‐like (DOT1L) axis. AMPK‐activated SIRT3 also delays endothelial senescence by upregulating telomere reverse transcriptase expression.^28^

Metformin's protective effects have been confirmed in both animal and human studies. Chronic low doses of metformin given to mice that had poor lipid‐clearing capabilities and age‐related atherosclerosis led to reduced subendothelial inflammation and pro‐inflammatory cytokines levels.^28^ Bovine aortic endothelial cells exposed to clinically relevant amounts of metformin had increased activities of nitric oxide synthase (eNOS), endothelium‐derived nitric oxide (NO), and AMPK, while no such effects were observed in AMPK knockout mice. NO and eNOS maintain vascular homeostasis and its integrity, supporting the vascular‐protective effects of metformin.^29^, ^30^ Treating 32 week‐old mice with metformin at 200 mg/kg per day for four weeks also partially reversed left ventricular dilatation caused by δ‐sarcoglycan deficiency: the hearts showed less fibrosis, less cardiomyocyte hypertrophy, and fewer degenerative subcellular changes. At the same time, there was increased autophagy, increased AMPK activity, and suppressed mammalian target of rapamycin (mTOR) phosphorylation.^31^ Diabetic veterans (mostly white male) who took metformin had lower CVDs and mortality risks compared with those who took sulfonylureas, and similar results were obtained in another clinical trial that compared glipizide and metformin.^32^, ^33^ Although in these studies it could not be determined whether the results were caused by the benefits of metformin or damages due to sulfonylureas or both, metformin's protective effects could be more ascertained in the UK Prospective Diabetes Study (UKPDS), in which metformin treatment conferred a significantly lower incidence of myocardial infraction (33%, *P* = .005) compared with dietary therapy for diabetic patients.^34^ Furthermore, several clinical trials and meta‐analyses have found metformin to decrease CVD risk for pre‐diabetic and non‐diabetic people as well.^35^, ^36^

## 4 TUMORS

The risk of fatal cancer development increases exponentially with age, and around 60% of cancers are diagnosed in people aged 65 or older. Activation of oncogenes and shutdowns of tumor suppressor genes result in reprogrammed energy metabolism and uncontrolled cell proliferation.^37^ Multiple studies have shown metformin to be effective against various cancers, including lung, breast, and colorectal cancer.^38^ Metformin delayed the first tumor onset by 22% and 25% respectively in female mice at the age of 3 and 9 months.^39^ Furthermore, metformin inhibited nicotine‐derived nitrosamine ketone (NNK)‐induced lung cancer cell proliferation in mice by decreasing the levels of circulating insulin and insulin‐like growth factor 1 (IGF‐1), which suppressed the insulin/IGF‐1 signaling (IIS) pathway and the downstream mTOR signaling pathway.^40^ In endometrial cancer cells, metformin significantly reduced the levels of Ki‐67, an indicator of tumor progression, topoisomerase IIα, associated with DNA instability, and phospho‐ribosomal protein S6 and phospho‐extracellular signal‐regulated protein kinase (ERK) 1/2, which are downstream targets of mTOR. Significantly increased AMPK and p27 (a cell cycle regulator) levels and subsequent cell cycle inhibition were also observed.^41^ H19 is found in almost all cancer cells. Genome‐scale DNA methylation profiling showed that tumor promoting pathway genes became repressed and genes involved in neuronal development, cell morphology, and intracellular communication were activated after metformin treatment. Interestingly, the H19 gene was also inactivated, suggesting that a feed‐forward response to continuously suppress H19 can be established by metformin.^15^ In addition, the 11 differentially methylated CpG sites mentioned earlier were related to multiple tumor‐related genes: SIX3 downregulation in lung cancer due to promoter methylation was rescued by metformin; POFUT2 is linked to glioblastoma and adenocarcinoma; MUC4 is implicated in pancreatic cancer; KIAA1614 is related to colon cancer; lastly, UPF1 is associated with genome stability. The differentially methylated regions revealed the gene EPHB1, whose under‐expression leads to gastric carcinoma and invasion of colorectal cancer cells, and SERP2, which is positively correlated with body mass index (BMI) and abnormal glucose tolerance as well as colorectal cancer. Pathway enrichment analysis found the unfolded protein response, which is involved in metformin‐induced apoptosis in acute lymphoblastic leukemia.^18^

In 2005, a case control study first discovered metformin reduced risk of cancer in diabetic patients.^42^ Compared with sulfonylureas, insulin, and other anti‐diabetic drugs, metformin conferred a significantly lower risk of cancer (Hazard Ratio (HR) 0.63, 95% Confidence Interval (CI) 0.53–0.75).^43^ Diabetic patients who took metformin also had 7% less chance of getting hepatocellular cancer for each incremental year they took metformin, and it was attributed to inhibited proliferation and cell cycle arrest induced by metformin.^44^ Nevertheless, evidence from randomized control trials has been largely inconclusive.^45^, ^46^ Additionally, a study compared metformin with rosiglitazone and sulfonylureas, and metformin did not reduce malignancy rates in people.^47^ Multiple meta‐analyses also did not support metformin reducing cancer incidence.^48^, ^49^ Work is still needed to resolve these inconsistencies.

## 5 NEURODEGENERATION

Alzheimer's Disease (AD) accounts for 60%–80% of dementia and affects up to one third of the population aged >65 years, making it the fifth leading cause of death globally.^50^, ^51^ Currently, no drug targets the development of AD. Instead, the impaired cognitive functions are treated with cholinesterase inhibitors and glutamate inhibitors to prevent the breakdown of acetylcholine and the over‐excitation of neurons.^52^, ^53^, ^54^, ^55^

Applying metformin to treat AD stems from the widely observed association between AD and T2DM. Treatment with metformin for eight weeks improved cognitive functions in 20 non‐diabetic patients.^56^ Nonetheless, data from 7086 dementia patients and matching number of healthy controls from the UK‐based General Practice Research Database (GPRD) concluded otherwise; diabetic individuals who had not used any drug (Adjusted Odds Ratio (AOR) 0.88, 95% CI 0.71–1.10) or those who had (AOR 1.03, 95% CI 0.90–1.19) had a similar risk of developing AD to individuals without diabetes (AOR 1). Furthermore, patients who had been exposed to various anti‐diabetic drugs and had received more than 60 prescriptions of metformin had higher risk of developing AD (AOR 1.71, 95% CI 1.12–2.60), which was attributed to the production of A‐β peptides, a hallmark for AD. However, the risk did not increase with the number of metformin prescriptions, and patients who had taken metformin exclusively did not have increased risk (AOR 1.00, 95% CI 0.55–1.81, >30 prescriptions).^57^ How metformin stimulated A‐β peptides production was explained on cell cultures of primary cortical neurons and N2a neuroblastoma cells expressing human amyloid precursor protein (APP): metformin upregulates β‐secretase, which cleaves APP into A‐β peptides. Intriguingly, metformin combined with insulin reduced A‐β peptide levels.^58^ In diabetes model mice, metformin attenuated total tau and phospho‐tau levels and activated c‐Jun N‐terminal kinases (JNK), a tau kinase. Metformin also attenuated the decrease of synaptophysin and preserved the neural structures but did not improve spatial learning and memory abilities.^59^ These studies together suggest that having taken metformin in the past may not reduce the risk of AD, but metformin used with insulin could be an effective short‐term treatment.

Parkinson's disease (PD) affects around 1% of people aged >60 years.^60^ Loss of dopaminergic neurons is characteristic of PD and leads to imbalance between dopamine and acetylcholine. Over‐activation of cholinergic system causes motor and cognitive disturbances. Hence, current PD drugs either provide more dopamine or reduce the amount of acetylcholine to restore the balance, working as a remedy instead of neuroprotective agents.^61^, ^62^, ^63^

Parkinson's disease, diabetes, and dementia share mitochondrial bioenergetics disorder and abnormal protein folding in their pathogenesis, and several studies have found metformin to alleviate PD. An analysis of a cohort of 800,000 people from the Taiwan National Health Insurance database showed that T2DM patients had 2.2‐fold risk of PD, and metformin‐inclusive sulfonylurea therapy reduced the risk (HR 0.78 relative to diabetes‐free, 95% CI 0.61–1.01).^64^ The reason has to do with metformin's ability to reduce α‐synuclein release, a component of the Lewy bodies and Lewy neurites that are characteristic of PD. Astroglial activation in damaged mice dopaminergic neurons increased α‐synuclein release. Metformin mitigated astroglial activation and promoted methylation of protein phosphatase 2A (PP2A), helping α‐synuclein dephosphorylation. AMPK activation by metformin also increased ATP production in mitochondria and restored mitochondria function. However, the dosage of metformin was also critical. Although metformin increased the levels of brain‐derived neurotrophic factor (BDNF) and glial cell line‐derived neurotrophic factor (GDNF), high dosage (400 mg/kg) killed all the mice.^65^, ^66^ In another study, tumor necrosis factor type 1 receptor associated protein (TRAP1) absence disturbed stress sensing in mitochondria and increased mitochondrial respiration, reduced mitochondrial membrane potential, and caused imbalance of nuclear and mitochondrial protein production, which were all rescued by metformin.^67^ In summary, metformin intervenes the pathogenesis of PD by preserving neurons, reducing inflammation, and protecting mitochondria functions. It is a promising treatment for PD, but further studies are still needed to understand the influence of dosage.

## 6 CONCLUDING REMARKS AND FUTURE PERSPECTIVES

Although current research gives promise to metformin as an anti‐aging drug, there are still concerns that need to be highlighted, and they apply not only to research into metformin but to other anti‐aging mechanisms and drug research as well.

First, despite the positive outcomes from many studies, it is not uncommon to find a change in dosage turning the result from life‐extending to life‐ending. When a low dose of metformin (0.1%) was given to middle‐aged male mice with their diet, their lifespans were extended by 5.83% on average, but a higher concentration (1%) became toxic.^17^ In another study, although metformin activated AMPK and suppressed lipid storage in fruit flies, their lifespan did not increase. At higher doses (25 and 50 mM) metformin reduced the survival rates. The authors reasoned the causes to be excessive starvation, disrupted intestinal fluid homeostasis, or metformin toxicity.^68^ In the PD study with mice models, although metformin increased BDNF and GDNF levels, the high dosage (400 mg/kg) killed all the mice.^66^ The issues with dosage along with physical and genetic differences between humans and animals make scaling the positive lab results for human use a tricky matter. In the study that showed metformin's beneficial effects for treating CVDs in mice, the dosage was 200 mg/kg, which is definitely not applicable to humans.^31^ Hence, conducting human clinical trials may be a more straightforward approach to finding a safe and effective dosage for human use. Encouragingly, when anti‐diabetic doses of metformin were given to 12 pre‐operative endothelial cancer patients and comparison of their tissue samples before and after the operation was made, the same effects observed in vitro were found—increased AMPK phosphorylation, decreased tumor cell proliferation, and decreased H19 levels.^15^

Another issue standing in the way relates to the side effects associated with chronic use of drugs. About 25% of patients treated with metformin have gastrointestinal side effects associated with the phenotype of organic cation transporter 1 (OCT1).^69^ Besides, chronic use of metformin can cause dose‐dependent vitamin B12 deficiency, increasing the risk for anemia and neuropathy.^70^, ^71^ Lactic acidosis has been reported as a side effect of metformin, but there have been controversies. In the study using diabetes model mice to study AD‐like brain changes, metformin did not further increase the serum lactate concentrations.^59^ Whether this holds true in non‐diabetic mice or humans is yet to be seen. The issues with side effects can be addressed in four ways. The first is to selectively take supplements, such as vitamin B12, to make up for the loss. The second is to reduce the dose and increase the interval between every dose, but more studies are needed to calibrate the balance between safety and anti‐aging potency. The third is taking a variety of anti‐aging drugs (also known as drug cocktail therapy), each with a very low dose, instead of taking only metformin, since the side effects are dose‐dependent. Although cultured cells and a mice study both showed metformin combined with insulin reduced A‐β peptide levels,^58^, ^59^ the GPRD study showed long‐term combined exposure to metformin and other anti‐diabetic drugs increased the risk of AD, while using only metformin did not show any difference.^72^ Therefore, this method requires further validation as well. The fourth way is to find analogs with fewer side effects. Currently, metformin has few available analogs with well‐studied and tolerated side effects. Phenformin and buformin, two biguanide drugs like metformin, were withdrawn from the market due to fatal lactic acidosis.^73^ Mito‐metformin, synthesized by adding a positively charged triphenylphosphonium group to metformin, showed 100‐fold to 1000‐fold anti‐proliferative effects on cancer cells depending on alkyl chain length, but how the dramatically improved potency will impact healthy cells is poorly understood at the moment.^74^

Future research should also work to elucidate how gender influences drug effectiveness. Metformin increased mean lifespan of female mice by 4.4% while decreasing that of male mice by 13.4%.^75^ Male pre‐diabetic patients who received metformin had a significantly lower coronary calcium score compared with control, while the female group did not.^36^ These studies have shown that gender influences drug effectiveness to varying extents, and studying drug‐hormone interactions could help find the reason.

Besides these issues, much more can be found about the genetic mechanisms that regulate lifespan and drug targets. APOE, a locus on chromosome 5q33.3, and FOXO3A are all well‐known longevity‐related genes. A genome‐wide association study (GWAS) could also identify five single nuclear polymorphisms (SNP) related to cerebellum aging.^76^ Although many positive outcomes have come from attempts to control DNA methylation with metformin, the full picture of epigenetic modifications has not been understood. Metformin treatment led to a combination of hyper, hypo, and non‐differentially methylated CpG sites, and this was due to a combination of direct and indirect effects. Hypermethylation of one site can change expression of a protein, and this can have downstream effects that alter methylation status of other sites.^15^ Understanding this complex network of interactions will not only promote further understanding of metformin but also help develop more anti‐aging measures targeting epigenetic modifications. In addition, SNPs in the human genome also affect the efficacy of drugs (Table 2). For example, rs2282143, located on SLC22A1, changes breast cancer cells' response to metformin by affecting the rate of the drug entering cells.^77^ A locus on chromosome 11 (rs11212617) is associated with the glycemic response to metformin.^78^ Three SNPs (rs8111699, rs11212617, rs9803799), which are located on LKB1, ATM and PRKAA2, have been identified as significant influencers on metformin therapy by affecting the AMPK pathway.^79^, ^80^, ^81^ As DNA sequencing becomes more convenient and accessible, an increasing amount of genetic data and research efforts will reveal many more such connections, and they can point to novel genes and drug targets or be used for precision medicine to improve current treatments. The advent of single cell sequencing technologies and multi‐omic profiling also opens the path to characterizing drug response down to the cell and tissue level to ensure the safety and effectiveness of treatment.

**TABLE 2:** A summary of major studies that have shown the effects of SNPs on metformin therapy

Study	SNP	Gene	Effect
Trilla‐Fuertes L (2018)^77^	rs2282143	SLC22A1	Affecting the efficiency of metformin entering cells
Lopez‐Bermejo A (2010)^79^	rs8111699	LKB1	Influencing both insulin sensitivity and metformin efficacy in hyperinsulinemic girls with androgen excess
Jablonski KA (2010)^80^	rs11212617	ATM	Affecting glycemic response to metformin
Zhou (2011)^81^	rs9803799	PRKAA2	Affecting generation of AMPK

The issues of dosage, side effects, sexual dimorphism, and genetic regulatory mechanisms all point to the need for large‐scale clinical trials. The Metformin in Longevity Study (MILES) involved 14 > 70 year‐old people who were randomized to take metformin and placebo in either order each for six weeks with a two‐week washout period in between. As the number of subjects was small and the duration short, MILES effectively revealed many transcriptomic and metabolomic changes in the muscle and adipose tissue.^82^ The Glucose Lowering In Non‐diabetic hyperglycaemia Trial (GLINT) is intended to evaluate the performance of metformin in reducing CVD risks by following 20,000 hyperglycemic but non‐diabetic patients for five years. A one‐year feasibility RCT enrolling 249 elderly, obese, and with high CVD risk (mean 28.8%, SD 8.5%) participants was concluded in 2018, and metformin improved several CVD risk indicators and decreased vitamin B12 levels. However, it also revealed problems such as a high rate of trial discontinuation (30% by six months).^83^ The Targeting Aging with Metformin (TAME) trial is a large placebo‐controlled trial that is designed to enroll 3000 subjects to test whether metformin delays age‐related diseases.^84^ The TAME trial received FDA approval in 2015, and after receiving all the required budget in 2019, it was set to start at the end of the same year. The TAME trial may make metformin the first approved drug for anti‐aging, but, more importantly, since it is not testing metformin against a single disease but a collection of age‐related ones, it establishes aging as a medical condition that can be intervened or treated instead of an irreversible process outside human control. The shift in the notion of aging will enable future anti‐aging clinical to trials proceed with much more ease.^85^

## ACKNOWLEDGMENTS

We sincerely appreciate the support from the Lars Blound institute of Regenerative Medicine in Qingdao. We thank Jessica Mar for her insightful comments in language revising and proofreading during the composition of the manuscript.

## CONFLICTS OF INTEREST

Nothing to disclose.

## AUTHOR CONTRIBUTIONS

TL, NC, and QF conceived the scope of the review. CW, BC, and QF conducted the literature research and drafted the manuscript. CW further revised the article. The final version is approved by all the authors.
